# Supplementary material for: The Effect of Circulating Zinc, Selenium, Copper and Vitamin K1 on COVID-19 Outcomes: A Mendelian Randomization Study
Source: Nutrients. 2022 Jan 6;14(2):233. doi: 10.3390/nu14020233 (PMC8780111; doi:10.3390/nu14020233)
Supplement: Supplementary file 1 [file nutrients-14-00233-s001.zip › Supplementary Methods.pdf]

Supplementary Methods

Exposure GWAS cohorts

Australia (QIMR) – Zn, Se, Cu (Evans et al. 2013)

The final cohort included 2,603 adults with matching genotype and phenotype data who participated in one of two studies on twins carried out at the Queensland Institute of Medical Research (QIMR).

The first study recruited twins born before 1964 who belonged to the Australian Twin Registry. Blood was collected from 1,134 men and 2,241 women between 1993-1996. Erythrocytes were used for elemental analysis. Cu, Se and Zn concentrations were measured by inductively coupled plasma mass spectrometry (ICP-MS) on a Perkin-Elmer Elan 5000 mass spectrometer (PerkinElmer, Inc., Wellesley, MA, USA) or a Varian UltraMass (Varian Inc., Palo Alto, CA, USA) and haemoglobin concentration was measured using the cyanmethemoglobin assay. Measurements were log-transformed and analysis batch, haemoglobin concentration and analytical quality control data were used as covariates to generate standardized residuals for GWAS analysis. 1,570 individuals out of 2,926 participants with elemental concentrations had also SNP genotyping data available. Illumina chips were used to determine genotypes with quality control and imputation procedure as previously described[1].
The second study took place in 2001-2005 and was a twin-family design, which included relatives of twin probands in the previous studies. Blood samples were collected from 8,396 people. Erythrocytes were used for elemental analysis. Cu, Se and Zn concentrations were measured by inductively coupled plasma mass spectrometry (ICP-MS) on Agilent 7500 system (Agilent Technologies, Inc., Santa Clara, CA, USA). Genotypes and standardized residuals were generated using the same method as in the first study. Phenotype and genotype data were available for 1,104 subjects.

Having removed duplicate participation of 71 individuals in both studies, the final set of 2,603 phenotype-genotype samples was arrived at.

UK (ALSPAC) – Se (Evans et al. 2013)

The Avon Longitudinal Study of Parents and Children (ALSPAC) recruited pregnant women resident in the Bristol area (United Kingdom) with due dates of 1 April 1991 to 31 December 1992. Maternal blood samples were obtained by midwives during the first appointment with the pregnant women. Se metal analysis was performed by the Center for Disease Control, Atlanta, GA in 2009–2010 using inductively coupled plasma dynamic reaction cell mass spectrometry (ICP-DRC-MS). Measurements were log-transformed and analysis batch was used as a covariate to generate standardized residuals for GWAS analysis.

A total of 10,015 women were genotyped using Illumina 660 quad SNP chip. SNPs were filtered to remove rare variants with <1% frequency, >5% missing data and markers which failed the Hardy-Weinberg equilibrium test. Individuals with non-western European ancestry were removed from the analysis, as were samples with

>5% missing data, unusual heterozygosity as well as close relatives. Following data cleaning, 8,340 individuals remained in the analysis, 2,874 of which also had the phenotype data available.

USA (CARDIA) – Se (Cornelis et al. 2015)

The CARDIA (Coronary Artery Risk Development in Young Adults) is a prospective study which recruited 5,115 adults (aged 18-30 years) of European and African-American ancestry in 1985-1986. The recruitment was done from community in Birmingham, AL; from selected census tracts in Chicago, IL and Minneapolis, MN; and from the Kaiser Permanente health plan membership in Oakland, CA.

USA (JoCo) – Se (Cornelis et al. 2015)

The JoCo (Johnston County Osteoarthritis Project) is a prospective study located in the townships of Johnston County, NC which enrolled older (age at least 45 years old) individuals of European and African-American ancestry following household enumeration in 1990. DNA collections were carried out at the first follow-up (1999-2003) and cohort enrichment (2003-2004) stage.

USA (NHS) – Se (Cornelis et al. 2015)

The Nurses' Health Study (NHS) is a prospective study of 121,700 female nurses resident in 11 large states and between ages of 30 and 55 when enrolled in 1976. Blood samples were collected from 32,826 participants between 1989 and 1990. Women selected for this study were previously part of case-control GWAS analyses for type 2 diabetes (T2D), coronary heart disease (CHD) or breast cancer (BrCa).

USA (HPFS) – Se (Cornelis et al. 2015)

The HPFS (Health Professionals Follow-up Study) is a prospective study of 51,529 male health professionals between ages of 40 and 75 which started in 1986. Blood samples were collected from 18,225 participants between 1993 and 1996. Men selected for this study were previously part of case-control GWAS analyses for type 2 diabetes or coronary heart disease.

Se toenail concentration (Cornelis et al. 2015)

Toenail clippings from all 10 toes were provided in the following years: 1982, 1983, 1986, 1987 (NHS, HPFS), 1987 (CARDIA), 1999-2003 (JoCo). Toenail Se concentrations were taken using neutron activation analysis at the University of Missouri Research Reactor between 2009 and 2011 for NHS and HPFS, 2006 and 2009 for CARDIA and 2004–2005 for JoCo. The samples represent a measure of Se exposure over approximately 1 year. Validation of measurements and laboratory equipment were undertaken using standard procedures. Individuals with toe-nail concentration of 2.0 µg/g were removed as such high concentration could come from contamination or excess ingestion of Se supplements.

Genotyping (Cornelis et al. 2015)

DNA extracted from blood samples was genotyped using the Affymetrix Genome-wide Human 6.0 array (NHS-T2D, HPFS-T2D, NHS-CHD, HPFS-CHD, CARDIA), Illumina Human-Hap550 array (NHS-BrCa) or Illumina 1M Duo array (JoCo). Details of genotyping and quality control for each population have been described previously[2–6]. In the CARDIA study, samples with more than 2% missing data were excluded. At a minimum, DNA samples with less than 90% completion threshold were excluded and SNPs with <95% call rates, rare SNPs (<2% frequency), as well as SNPs deviating from Hardy-Weinberg equilibrium were removed. Any samples of non-European ancestry were subsequently dropped. Imputation of markers to HapMap Phase 2 CEU reference panel was then carried out.

USA (FOS) – vitamin K_1 (Dashti et al. 2014)

The Framingham Offspring Study (FOS) is a community-based study among adult offspring and offspring spouses of the Framingham Heart Study original cohort. The study commenced in 1971 with 5,124 individuals enrolled. Fasting plasma or serum was collected between 1995-1998. In the current GWAS study, 1,607 individuals with genotype and phenotype data as well as dietary information available were included.

USA (Health ABC) – vitamin K_1 (Dashti et al. 2014)

The Health ABC study is a prospective study located in the metropolitan areas of Pittsburgh, PA and Memphis, TN which enrolled elderly (aged 70-79 years old) but community-dwelling and Medicare-eligible individuals of European and African-American ancestry between 1997-1998. Plasma samples were taken in 1998-1999 from 1,110 participants in the knee osteoarthritis case-control sub-study. 531 samples were eligible for measurement of phylloquinone due no warfarin use and European ancestry.

Vitamin K_1 plasma/serum concentration (Dashti et al. 2014)

Serum or plasma phylloquinone measurements were taken at the Vitamin K Laboratory at the USDA Human Nutrition Research Center on Aging at Tufts University in Boston, MA in 2012-2013. Reverse-phase high performance liquid chromatography (HPLC) followed by fluorometric detection was used. The laboratory participated in the international vitamin K external quality assurance scheme. The lower limit of detection was 0.1 nmol/L and samples with concentrations below that threshold were coded as 0.05 nmol/L.

Genotyping (Dashti et al. 2014)

Genotyping was carried out with Affymetrix 500k and MIPS 50k platform for FOS and Illumina 1M platform for Health ABC as described previously[7,8]. Rare SNPs with variant frequency of <1%, low call rate (<97%) and not in Hardy-Weinberg

equilibrium were excluded. Imputation of markers to HapMap Phase 2 CEU reference panel was then carried out.

Vitamin K_1 genetic instruments

We removed 4 vitamin K_1 SNPs from downstream processing. The first one was the exceptionally pleiotropic (associated with 74 traits in GWAS Catalog and 96 in PhenoScanner) and confounded (*p*-value close to 1 in two of alternative GWAS models) rs964184. Secondly, we excluded rs2108622 (along with rs12609820 in the same clump) which was also highly significantly associated with vitamin E concentration phenotypes. Lastly, we had to discard rs2192574 as it was unavailable in the outcome dataset and no proxy could be found.

Phenome-wide scan results

Out of known risk-factors for COVID-19 with evidence from previous MR analyses[9] – body mass index (BMI) for hospitalization and infection, smoking for hospitalization, height and low red blood cells (RBC) for infection, we found overlap with adiposity, height and RBC traits. One SNP (rs2769264, Cu) was associated with adiposity traits, while 5 with height (rs921943, rs10944 – Se; rs248381, rs17823744, rs2163813 – Se sensitivity analysis). We did not remove the Cu instrument (rs2769264), as it is the strongest and one of only 2 instruments for Cu nutrition, but we note that the Cu associations are by far the strongest at the locus (*p*-value = 2.63×10^{-20}) and adiposity related traits are only nominally associated (*p*-value > 1×10^{-7}). Furthermore, copper is known to play an essential role in fat catabolism[10,11] and so BMI is likely a case of vertical pleiotropy at this SNP, downstream from copper exposure, which does not violate MR assumptions. We were also cautious with SNPs related to height, as height is a famously highly complex polygenic trait[12], and higher COVID-19 reported infection rate in tall individuals currently lacks a plausible biological mechanism. It is causally not clear whether the impact of red blood cell number on COVID-19 infection is not possibly related to their micronutrient content of interest here, so SNPs associated with RBC phenotypes (rs1175550 – Cu, rs2120019 – Zn, rs1532423 – Zn) were also retained. Again, for all those instruments, stronger association was found for micronutrient content relative to RBC phenotypes, except for rs1175550, despite pronounced difference in respective GWAS power: *n*=2,603 for Zn and Cu, *n*= 173,480 for RBC phenotypes; these SNPs were also more weakly associated with other blood cell phenotypes.

References

1. Medland, S.E.; Nyholt, D.R.; Painter, J.N.; McEvoy, B.P.; McRae, A.F.; Zhu, G.; Gordon, S.D.; Ferreira, M.A.R.; Wright, M.J.; Henders, A.K.; et al. Common Variants in the Trichohyalin Gene Are Associated with Straight Hair in Europeans. *Am. J. Hum. Genet.* **2009**, *85*, 750–755, doi:https://doi.org/10.1016/j.ajhg.2009.10.009.

2. Laurie, C.C.; Doheny, K.F.; Mirel, D.B.; Pugh, E.W.; Bierut, L.J.; Bhangale, T.;

Boehm, F.; Caporaso, N.E.; Cornelis, M.C.; Edenberg, H.J.; et al. Quality control and quality assurance in genotypic data for genome-wide association studies. *Genet. Epidemiol.* **2010**, *34*, 591–602, doi:https://doi.org/10.1002/gepi.20516.

3. Jensen, M.K.; Pers, T.H.; Dworzynski, P.; Girman, C.J.; Brunak, S.; Rimm, E.B. Protein Interaction-Based Genome-Wide Analysis of Incident Coronary Heart Disease. *Circ. Cardiovasc. Genet.* **2011**, *4*, 549–556, doi:10.1161/CIRCGENETICS.111.960393.

4. Hunter, D.J.; Kraft, P.; Jacobs, K.B.; Cox, D.G.; Yeager, M.; Hankinson, S.E.; Wacholder, S.; Wang, Z.; Welch, R.; Hutchinson, A.; et al. A genome-wide association study identifies alleles in FGFR2 associated with risk of sporadic postmenopausal breast cancer. *Nat. Genet.* **2007**, *39*, 870–874, doi:10.1038/ng2075.

5. Lemaitre, R.N.; Tanaka, T.; Tang, W.; Manichaikul, A.; Foy, M.; Kabagambe, E.K.; Nettleton, J.A.; King, I.B.; Weng, L.-C.; Bhattacharya, S.; et al. Genetic Loci Associated with Plasma Phospholipid n-3 Fatty Acids: A Meta-Analysis of Genome-Wide Association Studies from the CHARGE Consortium. *PLOS Genet.* **2011**, *7*, 1–12, doi:10.1371/journal.pgen.1002193.

6. Sorge, R.E.; Trang, T.; Dorfman, R.; Smith, S.B.; Beggs, S.; Ritchie, J.; Austin, J.-S.; Zaykin, D. V; Meulen, H. Vander; Costigan, M.; et al. Genetically determined P2X7 receptor pore formation regulates variability in chronic pain sensitivity. *Nat. Med.* **2012**, *18*, 595–599, doi:10.1038/nm.2710.

7. Alan, H.; P., G.N.; B., M.M.; M., H.I.; Arne, P.; Thomas, I.; H.-Erich, W.; Thomas, M.; David, H.; B., H.F.; et al. A Common Genetic Variant Is Associated with Adult and Childhood Obesity. *Science (80-.).* **2006**, *312*, 279–283, doi:10.1126/science.1124779.

8. Murphy, R.A.; Nalls, M.A.; Keller, M.; Garcia, M.; Kritchevsky, S.B.; Tylavsky, F.A.; Newman, A.B.; Tranah, G.J.; Eiriksdottir, G.; Gudnason, V.; et al. Candidate Gene Association Study of BMI-Related Loci, Weight, and Adiposity in Old Age. *Journals Gerontol. Ser. A* **2013**, *68*, 661–666, doi:10.1093/gerona/gls227.

9. Initiative, C.-19 H.G. Mapping the human genetic architecture of COVID-19. *Nature* **2021**, doi:10.1038/s41586-021-03767-x.

10. Yang, H.; Ralle, M.; Wolfgang, M.J.; Dhawan, N.; Burkhead, J.L.; Rodriguez, S.; Kaplan, J.H.; Wong, G.W.; Haughey, N.; Lutsenko, S. Copper-dependent amino oxidase 3 governs selection of metabolic fuels in adipocytes. *PLOS Biol.* **2018**, *16*, 1–27, doi:10.1371/journal.pbio.2006519.

11. Krishnamoorthy, L.; Cotruvo, J.A.; Chan, J.; Kaluarachchi, H.; Muchenditsi, A.; Pendyala, V.S.; Jia, S.; Aron, A.T.; Ackerman, C.M.; Wal, M.N. Vander; et al. Copper regulates cyclic-AMP-dependent lipolysis. *Nat. Chem. Biol.* **2016**, *12*, 586–592, doi:10.1038/nchembio.2098.

12. Yengo, L.; Sidorenko, J.; Kemper, K.E.; Zheng, Z.; Wood, A.R.; Weedon, M.N.;

Frayling, T.M.; Hirschhorn, J.; Yang, J.; Visscher, P.M.; et al. Meta-analysis of genome-wide association studies for height and body mass index in ∼700000 individuals of European ancestry. *Hum. Mol. Genet.* **2018**, *27*, 3641–3649, doi:10.1093/hmg/ddy271.
