# Supplementary figures and images for: The Effect of Circulating Zinc, Selenium, Copper and Vitamin K1 on COVID-19 Outcomes: A Mendelian Randomization Study
Source: Nutrients. 2022 Jan 6;14(2):233. doi: 10.3390/nu14020233 (PMC8780111; doi:10.3390/nu14020233)

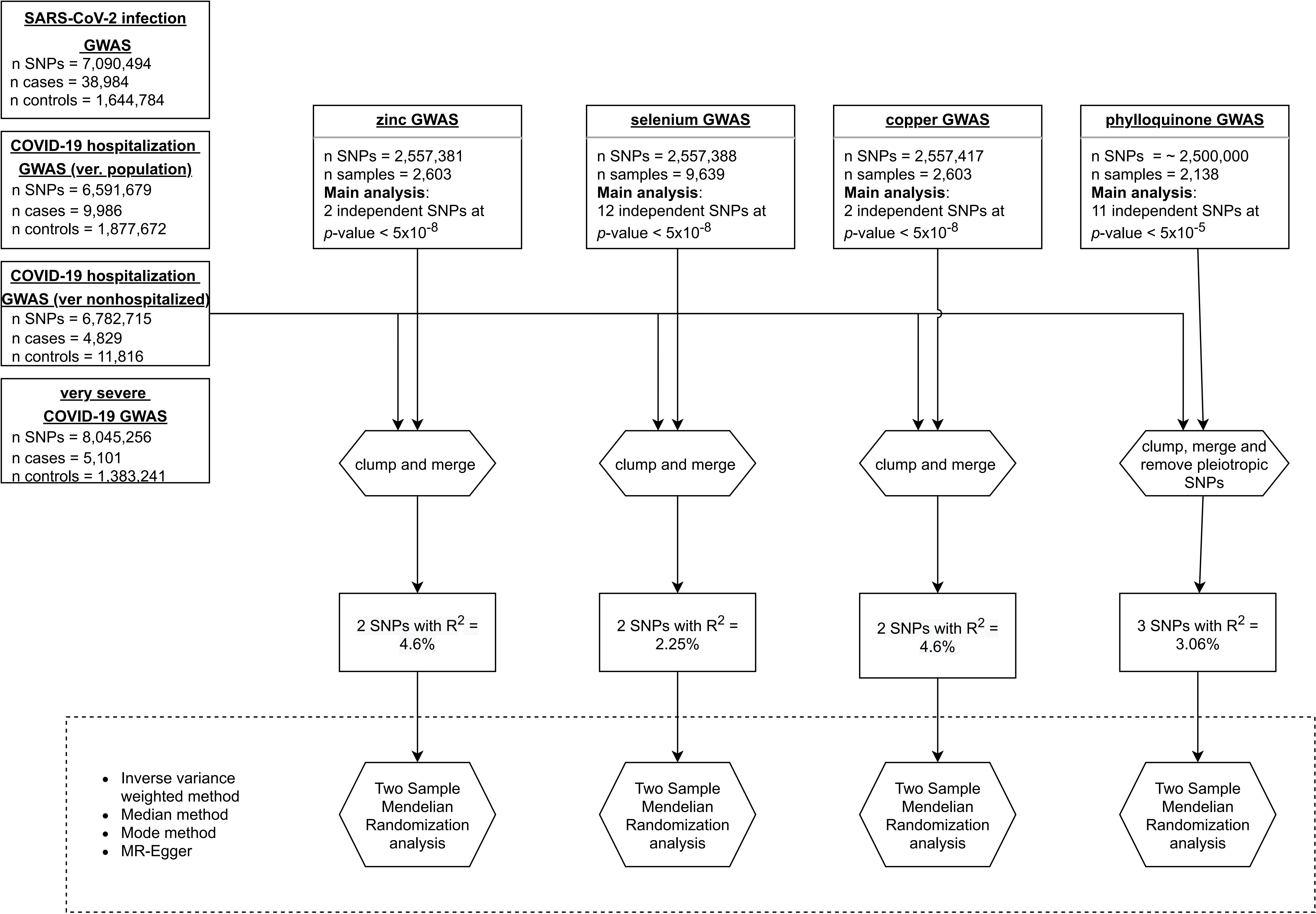

Supplement: Supplementary file 1 [file nutrients-14-00233-s001.zip › Figure S1.pdf]
